# Audit of the Ward Environment in an Inpatient Autism Unit

**DOI:** 10.1192/bjo.2025.10667

**Published:** 2025-06-20

**Authors:** Azmathulla Khan Hameed, Jhansi Seekulo

**Affiliations:** Cygnet Hospital Harrow, Harrow, United Kingdom

## Abstract

**Aims:** Creating an optimal ward environment for autistic inpatients is essential for their well-being and therapeutic progress. This audit aimed to assess the inpatient ward environment of two autism rehabilitation wards – Spring Center (a locked rehabilitation ward) and Spring Wing (an open rehabilitation ward) – against the Gold Standard Environmental Standards for Learning Disability (LD) and Autism Spectrum Disorder (ASD) inpatient hospitals, as well as the Quality Network for Learning Disability (QNLD) standards.

**Methods:** A structured questionnaire was developed based on gold-standard guidance for autism inpatient wards. The audit was conducted by a Staff Grade Doctor and a Specialist Occupational Therapist (OT), who inspected both wards, interviewed staff and patients, and evaluated adherence to 22 key environmental standards. The OT’s input was crucial in assessing the sensory needs of autistic individuals.

**Results:** Out of the 22 assessed parameters, both wards failed to meet 7 critical requirements, including:

Lack of consultation with autistic individuals regarding the design and assessment of sensory spaces.

Absence of active patient and family feedback regarding the ward environment.

Insufficient autism and sensory sensitivity training for all staff, including non-clinical members.

Lack of soft furnishings and carpets to reduce background noise.

No structured process to identify and minimize strong odours in patient areas.

Limited bedding options catering to individual sensory preferences.

No use of unscented cleaning and personal-care products.

The remaining 15 parameters were met in both wards. The findings were shared with the ward manager, hospital manager, and medical team, with plans to present them in a clinical governance meeting to develop a business case for environmental improvements.

**Conclusion:** The audit identified several areas requiring immediate attention to enhance the sensory environment and overall ward quality for autistic inpatients. It also highlighted the importance of specialized spaces and therapy rooms tailored to sensory needs. A re-audit is planned in six months to assess the implementation of recommendations and ensure continued improvements in the ward environment. An autism ward environment should be designed to be calm, low-sensory, and predictable, with features like soft lighting, quiet spaces, minimal noise, clear visual cues, and a consistent routine to minimize sensory overload and create a therapeutic space for autistic individuals who can be easily distressed by overstimulation in a typical hospital ward; this often includes designated quiet areas, muted colours, and staff trained in autism-specific communication strategies.

